# LiDAR-Based System and Optical VHR Data for Building Detection and Mapping †

**DOI:** 10.3390/s20051285

**Published:** 2020-02-27

**Authors:** Silvia Liberata Ullo, Chiara Zarro, Konrad Wojtowicz, Giuseppe Meoli, Mariano Focareta

**Affiliations:** 1 Engineering Department, University of Sannio, 82100 Benevento, Italy; 2 Faculty of Mechatronics and Aerospace, Military University of Technology, 00-908 Warsaw, Poland; 3 Mapsat, 82100 Benevento, Italy

**Keywords:** LiDAR technology, 2D LiDARs, 3D LiDARs, optical VHR data, WorldView-2, environmental monitoring, analysis and classification, building feature extraction, Object-Based Image Analysis (OBIA)

## Abstract

The aim of this paper is to highlight how the employment of Light Detection and Ranging (LiDAR) technique can enhance greatly the performance and reliability of many monitoring systems applied to the Earth Observation (EO) and Environmental Monitoring. A short presentation of LiDAR systems, underlying their peculiarities, is first given. References to some review papers are highlighted, as they can be regarded as useful guidelines for researchers interested in using LiDARs. Two case studies are then presented and discussed, based on the use of 2D and 3D LiDAR data. Some considerations are done on the performance achieved through the use of LiDAR data combined with data from other sources. The case studies show how the LiDAR-based systems, combined with optical Very High Resolution (VHR) data, succeed in improving the analysis and monitoring of specific areas of interest, specifically how LiDAR data help in exploring external environment and extracting building features from urban areas. Moreover the discussed Case Studies demonstrate that the use of the LiDAR data, even with a low density of points, allows the development of an automatic procedure for accurate building features extraction, through object-oriented classification techniques, therefore by underlying the importance that even simple LiDAR-based systems play in EO and Environmental Monitoring.

## 1. Introduction

An efficient exploration of the surrounding environment is becoming the key point for the future world where human functionalities are going to be substituted by automatic or semiautomatic systems. Self-driving cars and charging robots for electrical cars are just some examples where the knowledge of the space in which to move becomes essential for the success of complex operations. In this context, Light Detection and Ranging (LiDAR)-based systems are going to play an increasingly more important role because of their higher capability to discriminate smaller objects with respect to other active systems, such as RAdio Detection And Ranging (RADAR) systems. In fact, LiDAR is an active object detection method similar to RADAR, but it makes use of other parts of the electromagnetic spectrum, predominantly infrared light rather than radio waves. LiDAR measures reflected light emitted by a laser, which has a much smaller wavelength than radio signals, and therefore its capability to discriminate smaller objects is much higher, and consequently also its capability to solve the navigation problems.

Since the 1990s LiDAR technology spread and continued to search for ts commercial use, until its rapid growth in 2010s. In the last decade, it has found an increasing use in combination with other data sources for land surface cover analysis and classification [1,2,3,4,5,6,7,8].

An extensive additional list of references, highlighting the main LiDAR characteristics, its advantages and disadvantages, and the different fields of applications with several case studies, can be found in [9,10,11,12,13], showing how LiDAR has contributed significantly in the EO and Remote Sensing fields. The above references can be regarded as useful guidelines for researchers interested in using LiDARs.

In this paper, we aim to further extend the analysis of LiDARs systems, by enriching the list of references, and proposing a classification algorithm, showing the performance that can be achieved through the use of LiDAR data, when combined with data from other sources, in exploring external environments and extracting building features from urban areas.

Relevant literature about the building detection with the employment of LiDAR can be found in [14], where the most important articles for building extraction are listed, by highlighting the approaches and the auxiliary sources used in combination with LiDAR data. Further important literature about the building detection, based on the fusion of LiDAR data with optical remote sensing imagery, can be found in [15,16,17,18].

Although there are studies that have concerned the building detection, by combining LiDAR data and optical imagery, the use of OBIA, in combination with optical satellite remote sensing data and low density LiDAR point cloud on extended areas, is not common in a simple LiDAR-based system version. For instance, the review in [14], summarizing the LiDAR point cloud density and covered areas for the most relevant papers, highlights that the dataset size is always less than one km${}^{2}$, and in the few cases in which it is greater than one, also the point cloud density increases, far beyond the unity. In this paper, we applied the proposed algorithm to an area of 5.3 km${}^{2}$, after testing it on a smaller area of only 200 square meters, with a LiDAR point cloud density equals approximately to 1.5 points per square meter (ppsm). Note that, although the work in [14] was published in 2015, if we look for other applications of the OBIA technique, in recent years, in similar conditions, no other results are comparable with those reached with the proposed algorithm, where such a low LiDAR point cloud density has been used in combination with optical imagery on wide urban areas. Moreover, the transferability from the tested area to the wider area has been carried out without any modification of the proposed algorithm.

Two Case Studies, based on the use of 2D and 3D LiDARs (2-dimensional and 3-dimensional LiDARs) are presented and analyzed in an extensive way.

The Case Studies show how the LiDAR-based systems succeed in improving the analysis and monitoring of specific areas of interest, specifically how LiDAR data help in exploring external environment and extracting building features from urban areas.

In particular, the case study of Lioni demonstrates that the use of the LiDAR data, even with a low density of points, allows the development of an automatic procedure for accurate building feature extraction, through object-oriented classification techniques, therefore by underlying the importance that even simple LiDAR-based systems play in EO and Environmental Monitoring. In the second case study, a measurement campaign has been carried out to test among others the 3D LiDAR MRS 6000 by the SICK company, a new device very useful for research purposes, resulting in an efficient tool for monitoring external environments, made by buildings, trees, roads, and other objects.

## 2. The LiDAR System

LiDAR is an active remote sensing technique used for performing high-resolution topographic surveys that operates in the visible or near-infrared regions of the electromagnetic spectrum. The survey could be carried out in flight (data collected using drones or airplanes), in space (data collected using satellites), or land-based (data collected from the ground). The very high-speed data acquisition and high ground resolution are the main characteristics of a LiDAR system [19]. The light pulse emitted by the transmitting device is reflected by a target to the sensor, and the system calculates the relative distance between them. By scanning a surface of interest, a cloud of points is created, that discriminates the points relative to the ground (represented through a Digital Terrain Model (DTM)) and those relative to the “objects” on the ground (represented through a Digital Surface Model (DSM)). By measuring the vegetative cover, and penetrating up to the ground, information is obtained on the altitudes with a centimetric accuracy. Many examples of LiDAR applications can be examined, for instance, from the punctual morphology representation of hydrogeological hazard areas, to the urban and infrastructure modeling and planning, to the design of power and energy distribution lines, and inventory and management of forests [9,10,11,12,13]. Moreover, the combination of LiDAR information with data from other sources can greatly help in improving the features’ extraction and classification for Earth Monitoring and Remote Sensing as highlighted in [20] and discussed later in this paper.

In the most common cases, the LiDAR sensor synchronously scans the distance to the obstacles in one plane. The result of a single scan is therefore a cloud of points in one plane only, and the LiDAR in this case will be referred to as a 2D LiDAR scanner. It is the most often used device in obstacle detection systems [21] and simultaneous localization and mapping (SLAM) [22]. It is then possible to obtain the third dimension by using a 2D LiDAR scanner, and, for example, by making the sensor’s head move in the direction perpendicular to the scanned plane.

LiDARs can be also mounted on the top of a quadrotor, above the rotors line. The results are merged with the current height values and in this way the number of dimensions can be extended, if necessary, for the measurements acquisition. LiDAR-based systems can be therefore used also in vertical take-off and landing (VTOL) of Unmanned Aerial Vehicles (UAVs), by making these operations completely autonomous. An example of an autonomously flying system is that developed at the University of Warwick [23] for outdoor applications, where a LiDAR on the top of an Unmanned Aerial Inspection Vehicle (UAIV) creates a 3D map of surroundings and it allows the UAIV to return to the starting point by following the same path. Another interesting work for vertical take-off and landing (VTOL) of UAVs is presented in [24] for indoor applications. Another example similar to this latter is given by the Hokuyo UGR-4LX LiDAR, which has been integrated with a switching mechanism and provides an ordinary 2D LiDAR with the 3D LiDAR features. The function of sweeping the scanning planes can be embedded inside the module of a 2D LiDAR by simulating what a 3D LiDAR does: scanning a few planes simultaneously. In this way, a 3D point cloud is provided by a 2D LiDAR, without external mechanical elements [25].

Other examples of laser-based systems, mounted on UAVs, are those where aerial photogrammetry solutions need to be employed [26]. In this case, the use of LiDARs allows the realization of simple visual navigation systems able to monitor large areas of interest with a high-speed data acquisition and a high ground resolution. One of the main concerns with UAVs is that fast movements in air require good performances in avoiding obstacles. A very interesting work has been done and presented by the Massachusetts Institute of Technology in [27] to this end. A collision avoidance system is realized, based on a visual alert that is delivered to an operator when the UAV is approaching an obstacle and the warning is computed according to data from a laser range scanner. Therefore, LiDARs demonstrate to give UAVs a large and important contribution for navigation.

As above introduced the so-called 3D LiDARs are laser sensors integrated with synchronously moving platforms for efficient performing of a scan. Realistically, it is the next step of the single-layer sensors mounted on moving stands of a 2D LiDAR module or embedded inside. In a “pure” 3D LiDAR, instead, the sensor can acquire data points simultaneously in many layers. Data acquisition is faster and more comfortable and results into a broader number of possible applications. Robotics seems to be the main beneficent of the rapid development of 3D LiDARs [28,29,30] and this is in accordance with initial observations on the significant role that LiDAR-based systems are going to play for an efficient exploration of the surrounding environment, to allow human functionalities to be substituted by automatic or semiautomatic systems. Other examples of important applications for 3D LiDAR-based systems can be found in [13,31,32,33], where 3D LiDAR data are used in combinations with cameras and radars for the indoor exploration of inside tunnels, underground mines, and caves.

## 3. Environmental Monitoring and Extraction of Features in Urban Areas

Exploration of the environment and its knowledge must be timely and precise to be efficient. Understanding urban dynamics, growth, and changes, brought by the urbanization, is fundamental for managing the land resources and providing services responding to the requests, in these rapidly changing environments [34]. Moreover, accurate and timely information on the coverage of the urban soil is essential for the government policies. However an urban environment is extremely complex and heterogeneous, typically composed of built structures (buildings and transport areas), various vegetation coverings (parks, gardens, and agricultural areas), areas of bare soil, and water bodies.

Remote sensing resources come to help in this regard, especially VHR images and LiDAR data, along with new analysis techniques, that allow to greatly improve mapping and classification of urban areas.

Among the different classification techniques, OBIA has attracted significant attention in recent years. Specifically, OBIA is a technique in which the semantic information is not enclosed in the single pixel but is found in an image-object, or groupings of pixels, that have similar characteristics, such as color, texture, and brightness. The aim of this technique is, therefore, to determine the specific characteristics of an object, its geometric, structural and relational features, with the corresponding thresholds for classifying different objects in the image. Numerous studies have shown that environmental monitoring and extraction of features in urban areas highly benefit when OBIA is jointly used with LiDAR [35,36,37,38,39]. In all these papers, an object-oriented approach has been applied for urban cover classification, using LiDAR data. The difference is in the parameter selection or in the number of classes, that can refer for instance to building, pavement, bare soil, fine textured vegetation, and coarse textured vegetation, resulting in a knowledge base of rules, potentially applicable to other urban areas. In the last paper, [39], some considerations on shadows affecting the areas under analysis, are also given, by showing how this may limit their correct classification.

In this paper, as already underlined in the introduction, we aimed to show how the LiDAR-based systems succeed in improving the analysis and monitoring of specific areas of interest, specifically, how LiDAR data help in exploring external environment and extracting building features from urban areas.

Two Case Studies are presented, and, in particular, the Case Study of Lioni (Avellino, Italy) demonstrates that LiDAR data, even with a low density of points, used in combination with VHR satellite imagery, allow the development of an automatic procedure for accurate building feature extraction, through the OBIA technique, therefore by underlying the importance that even simple LiDAR-based systems play in EO and Environmental Monitoring, whereas in the second case study, the use of a new 3D LiDAR device is discussed and tested through a measurement campaign carried out at the premises of Military University of Technology (MUT) in Warsaw, Poland.

The work done to this end will be presented in the next sessions.

## 4. Extraction of Building Features from LiDAR Data and WorldView-2 Images Through OBIA

This session is going to highlight how the combined use of LiDAR data and the VHR optical satellite WorldView-2 images, processed through OBIA, can produce a detailed map of the buildings in an urban area.

Moreover, this specific case study has been chosen with the aim of presenting a simple and precise workflow for building feature extraction, to provide decision makers with a tool capable of producing information related to the territory and useful to different needs. The next sub-sessions will describe the proposed algorithm.

### 4.1. Methods and Data

#### 4.1.1. Study Areas

This Case Study focuses on the urban areas of Lioni, a municipality in the province of Avellino (AV), in the Campania region, located in the south of Italy. Lioni is located 550 meters above sea level (expressed at the point where it is located the Municipal House). The geographical coordinates of Lioni are Latitude $40^{\circ }$$52^{\prime }$ North and $15^{\circ }$$11^{\prime }$ East.

The proposed algorithm was first applied to an area of Lioni, chosen randomly as tested area and extended approximately 200 m${}^{2}$.

Later, the same algorithm was applied to the whole area of Lioni, covering 5.3 km${}^{2}$, to verify its reliability and transferability. Results demonstrate the transferability of the proposed algorithm, without any parameter modification, as shown ahead in this paper.

**Figure 1:**
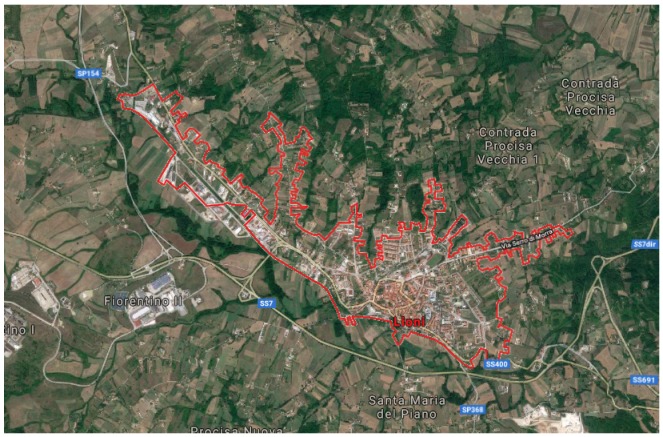
Lioni: Immages@2019 Google, Immages@2019 CNES/Airbus, Landsat/Copernicus, Maxar Technologies, Cartographic data (October 2019).

The town of Lioni and its surrounding area are shown in the Figure 1, where the reference Google Map has been retrieved in October 2019.

#### 4.1.2. Datasets

**Figure 2:**
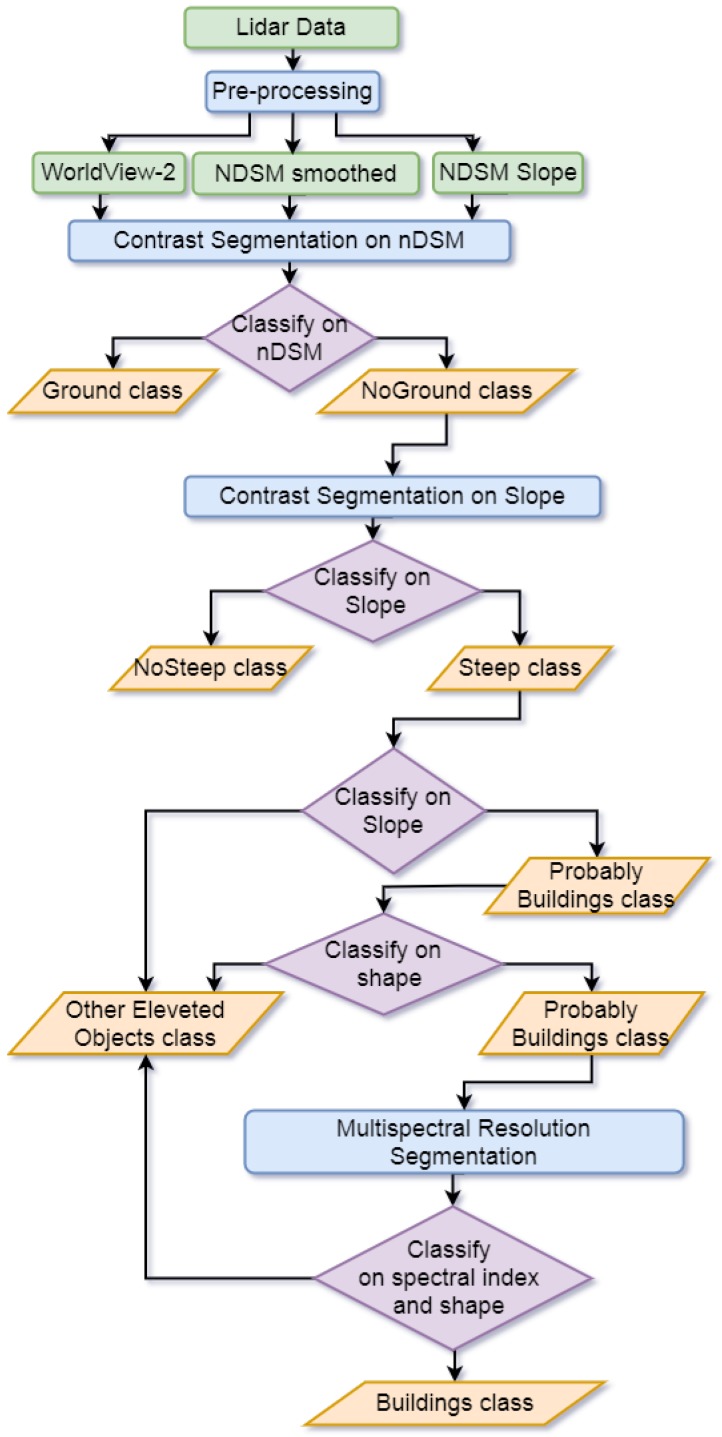
Workflow of the developed rule-based classification algorithm.

The proposed classification algorithm is shown in the Figure 2, where LiDAR data, WorldView-2 images, the Regional Technical Chart (CTR), and orthophotos are the inputs, as discussed briefly in the next paragraphs.

**Figure 3:**
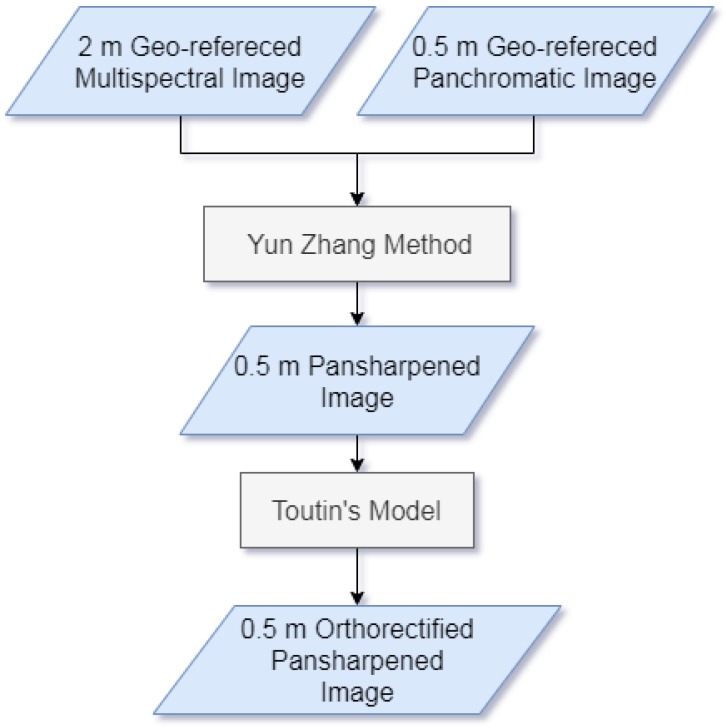
Flowchart for WorldView-2 image preprocessing.

- **LiDAR data**
  The Digital surface model (DSM) and the Digital Terrain Model (DTM) of Lioni were acquired from LiDAR dataset, property of the Italian Ministry for the Environment and the Protection of the Territory and the Sea, in conjunction with the Extraordinary Plan of Environmental Remote Sensing (EPRS-E) [40]. The acquisition year dates back to 2011 and the point cloud density is approximately 1.5 ppsm.
  Some considerations must be made about the ppsm of LiDAR data. Some case studies, presented in the literature, highlight the difficult to provide accurate models for buildings when low-density LiDAR data are used [41,42]. For example, in [41] the authors showed that is necessary a density point cloud higher than 5 ppsm in order to have better classification results. However, the data provided free of charge in many databases often cover vast areas but they have a point cloud density below 5 ppsm (such as the database of the Italian Ministry in the context of EPRS-E). Therefore, it is necessary to develop a specific strategy for features’ extraction over large areas with this type of data. Furthermore, the transferability of the strategy must also be carefully analyzed and discussed when these data are used.
- **WorldView-2 image**
  The WorldView-2 image on the area of Lioni was acquired by the satellite on 5 June 2017 at an angle of $16^{\circ }$, with cloud cover equal to 1%. The WorldView-2 image includes a panchromatic image with eight multispectral bands (Coastal Blue, Blue, Green, Yellow, Red, Red Edge, Near-Infrared-1, and Near-Infrared-2).
  The data were purchased by Mapsat (the company involved as a partner in this research) within the project “Asbesto 2.0”, and downloaded from the DigitalGlobe image archive, as a standard Ortho-Ready product projected on a plane with a UTM projection (Universal Transverse of Mercator) and a WGS84 datum. WGS84 is the abbreviation for World Geodetic System 1984, a geodesic, worldwide geographic coordinate system.
  A pan-sharpening and orthorectification procedure was performed by Mapsat. The image was pan-sharpened using the algorithm developed by Yun Zhang at the University of New Brunswick, in New Brunswick, Canada [43]. The pan-sharpening was performad to “transfer” the geometric resolution of the panchromatic datum, 0.5 meters (m), to the multispectral datum (with a 2 m resolution), to generate a final image with a resolution of 0.5 m, and improve the spatial information associated with the different bands. The orthorectification was applied using the using the CE90 standards [44] and the rigorous Toutin’s model [45], in accordance with the UTM 33 projection system, to have an error less than 4 m.
  The flowchart in the Figure 3 presents the main processing steps, as above described.
- **Regional Technical Chart (CTR) and orthophotos**
  To validate the results from the proposed algorithm, both the CTR of the Campania region and orthophotos, obtained under the license granted by the Agricultural Dispensing Agency of the Ministry of Agricultural and Forestry Policies, have been used. This is usually done, because the available public data are often not recent. In this case, the CTR dated back to 2004, at a scale of 1:5000, and the orthophotos were instead acquired in 2011, at a scale of 1:10,000 [46]. Therefore some buildings present in the orthophotos were not present in the CTR, because of new construction. On the other hand, some buildings in the CTR were demolished before the orthophotos survey, and therefore were not present in the 2011 data. The validation process will be discussed later in the paper.

#### 4.1.3. Processing LiDAR Data

**Figure 4:**
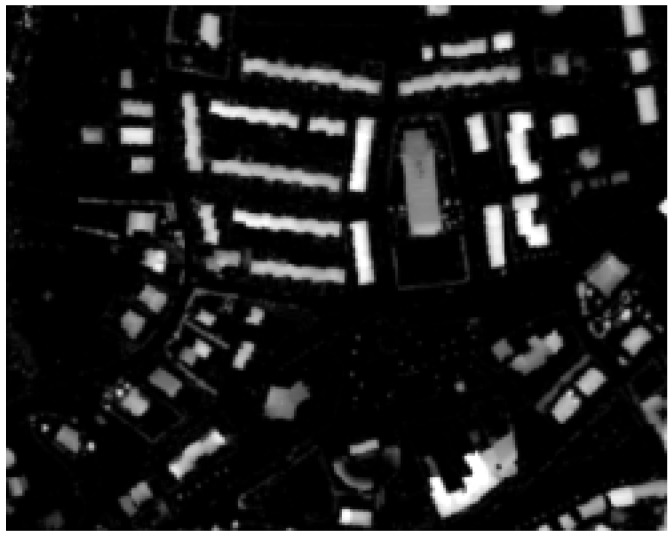
The normalized Digital Surface Model (nDSM) smoothed.

**Figure 5:**
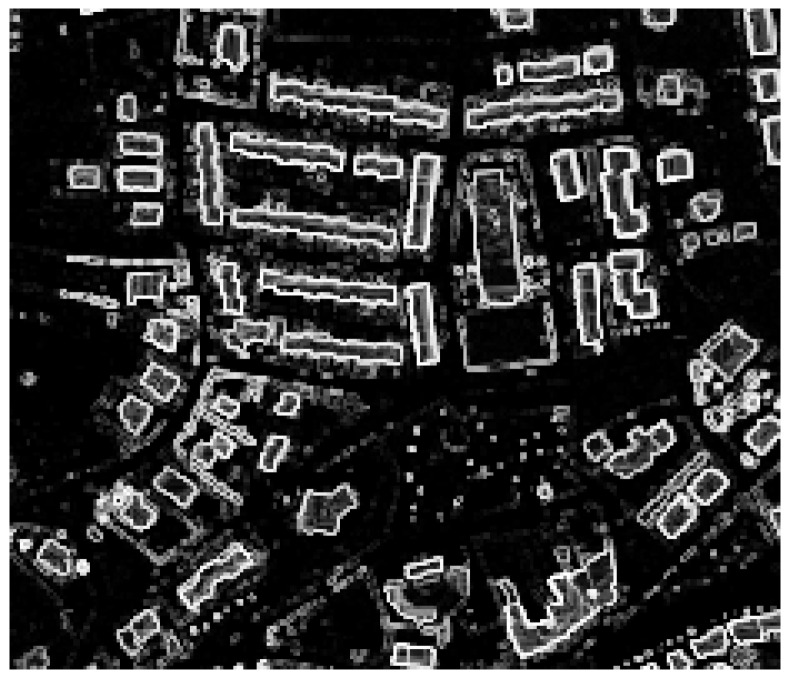
The slope map.

**Figure 6:**
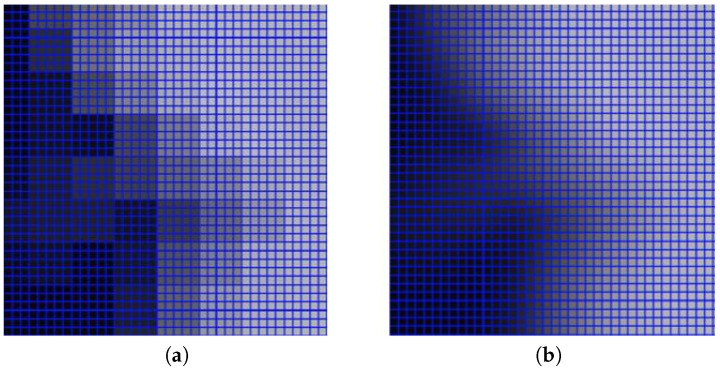
Pixels of the WordView-2 image before (**a**) and after the smoothing (**b**). Courtesy of the authors of [50].

The LiDAR DSM and DTM were processed in a QGIS enviroment [47], to generate three separate raster datasets: a normalized DSM (nDSM), a nDSM smoothed (Figure 4), and a slope map (Figure 5). A nDSM is generated by subtracting the DTM from the DSM, and it is necessary to identify high-rise objects in a DSM. High-rise objects in fact have a local nature due to the topography of the terrain surface [16]. Therefore, to eliminate the effects of topography from the DSM, the latter must be normalized [48]. Moreover, to get a better image quality for the nDSM, a smoothed nDSM is obtained by using a complex interpolation method, such as the *cubic B spline*. The objective of this method is to make the interpolation curve more smoothly and the image edges more defined. The smoothing operation is also necessary to adapt the geometric resolution of the LiDAR data (1 m) to the geometric resolution of the WordView-2 image (0.5 m), for matching the information from different data sources. Before the smoothing, groups of neighboring pixels in the WordView-2 image correspond to the same elevation (Figure 6a); after the smoothing, a different elevation is associated to each pixel (Figure 6b). In addition, as also shown in the figure, the magnified image, obtained by using the *cubic B spline* interpolation method, has no longer the so-called *saw tooth phenomenon* [49].

Processing the smoothed nDSM is also necessary in order to obtain information about the slopes of the objects present in the image under analysis. The slope map is calculated from the smoothed nDSM using the Zevenbergen Thorne method [51], implemented in QGIS. This method allows to have greater detail in the calculation of the curvature and is preferable with respect to other algorithms, like, for instance, the “Horn method”, in those applications where the processing of numerical data requires an exact definition of the parameters [52].

The slope represents a very important information, since it allows all buildings to be discriminated in the area of interest. In fact (with some exceptions in specific cultures which, for instance, tents are used for dwellings) all above ground artificial objects provide a strong and sudden change in height, forming a crisp edge around them [53]. Figure 4 and Figure 5 show, respectively, the nDSM and the slop map. The processing of LiDAR data, carried out and described above, has represented the starting point for the development of the proposed classification algorithm, used for the extraction of buildings’ features in the urban areas.

**Figure 7:**
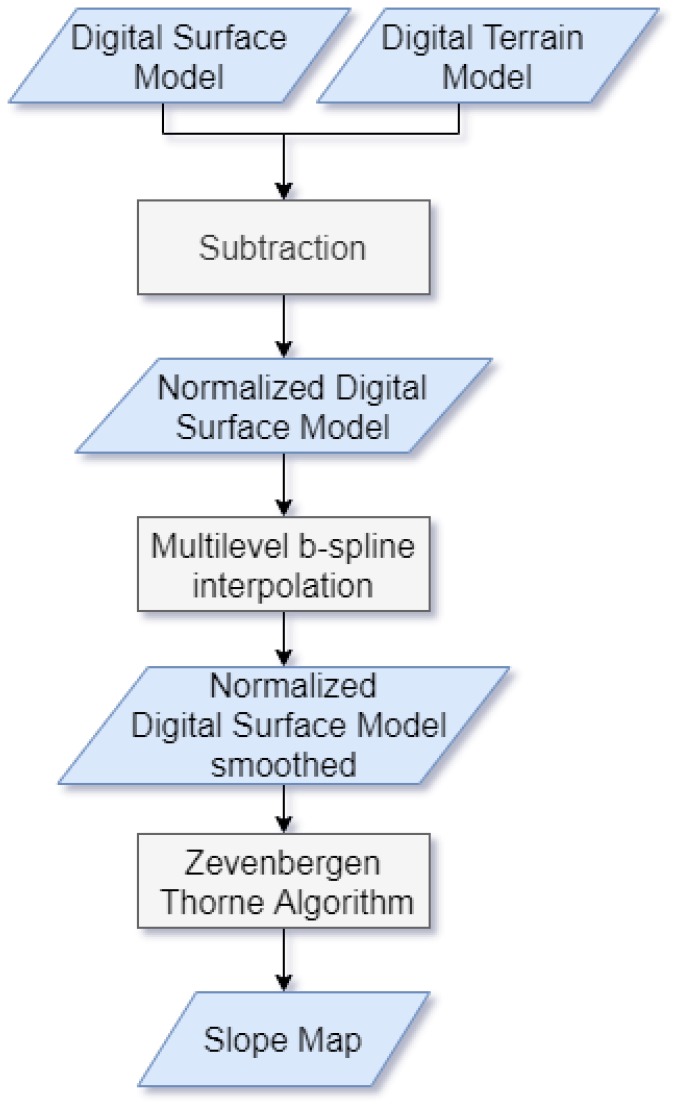
Flowchart for preprocessing LiDAR data.

The flowchart in the Figure 7 summarizes the main processing steps above described.

#### 4.1.4. Object-Based Classification Method

In recent years, OBIA has proved to be a very efficient method for classifying high-resolution images, because, as already highlighted in the paper, this technique relies on semantic information not enclosed in the single-pixel, but in an object of the image, that is in groups of pixels that have similar characteristics, such as color, texture, brightness.

The main steps in the development of the rule set for achieving the object-based classification are (1) the segmentation and (2) the feature extraction and classification.

The segmentation divides the image into separate and homogeneous regions (objects). The particularity of this operation is to use not only the spectral characteristics, but also the geometrical, structural and relational properties of an object. After the segmentation, the pixels of the image are grouped and, consequently, much information is available for each object (for example the spectral signature, the shape, and the size or the context), and in addition, features concerning the interrelations between the objects will also be available. All these attributes can be applied and combined for the development of a rule set to classify the objects in the scene of interest.

The feature extraction is the next step necessary to detect the objects of interest. As each object is characterized by particular features that distinguish it from other objects in the image, the feature extraction works in identifying these characteristics.

The rule-set creation is based on the analyst’s knowledge of the spatial, spectral, textural, and elevation characteristics of each feature. For instance, buildings have a different elevation pattern with respect to roads, roads are elongated compared with other features, trees have a coarser texture than grass, and various roof materials vary in texture and spectral characteristics. This type of human knowledge and reasoning is applied to the OBIA processing to separate the targeted objects from unwanted features [54]. In the rule-based classification phase, once the attributes have been chosen, thresholds must therefore be defined, on the basis of which objects are assigned to each class. In this case, the characteristics, chosen for the building extraction, belongs to the following three main categories: Average band values, Geometric features, and Spectral Indexes, summarized in the following Table 1.

The software used for the classification has been eCognition Developer, a product developed by Trimble for the analysis and interpretation of images, which allows semi-automatic information to be extracted, through an object-oriented classification [55].

To avoid burdening the manuscript, the rules adopted for the building extraction have been collected in an Appendix A, and numerical values are presented in the Appendix A, as well. They refer mainly to the workflow shown in the Figure 2. Note that the choice of the specific features for the proposed algorithm belongs to the authors’ expertise, based on the state-of-the-art knowledge and good sense and sound judgment, developed in practical matters related to the image analysis. Moreover, it must be underlined that, among the features shown in Table 1, some are more important than others. In fact, nDSM and slope, obtained from LiDAR data, represent the starting point for the development of the proposed classification algorithm.

The development of the set of carried out rules is, in fact based on the concept that the buildings are characterized by a sudden change of elevation at their edges and show a very different height, when compared to the surrounding environment. Therefore, for the extraction of buildings, the use of the information from the nDSM smoothed raster and the nDSM slope raster, previously described, is particularly important. Regarding other features, like, for instance, the Normalized Difference Vegetation Index (NDVI), the latter has been identified as a needful index for distinguishing vegetation, but this does not mean that other indexes cannot be added to improve the classification.

Extending the number of possible indexes can certainly represent a future development of this work.

### 4.2. Results Analysis and Accuracy Assessment

**Figure 8:**
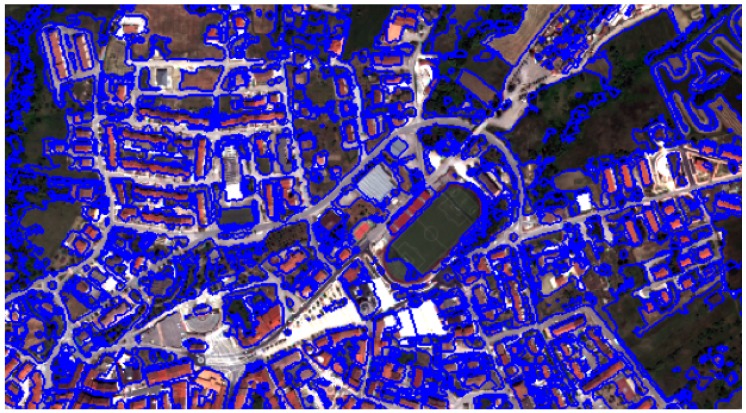
Result of the contrast segmentation for a first area of Lioni.

**Figure 9:**
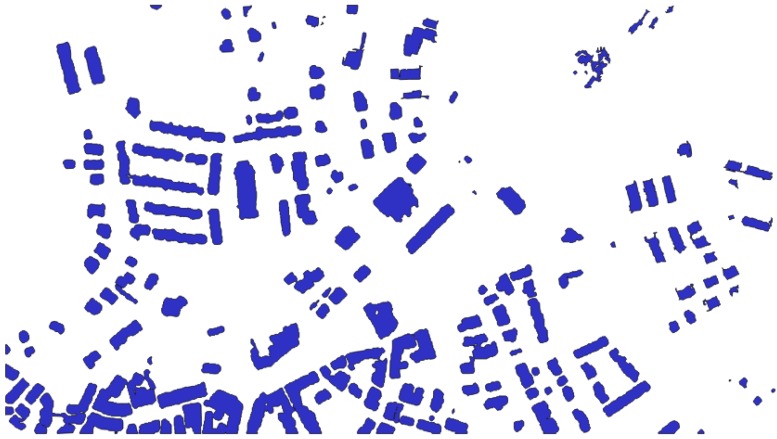
Result of buildings extraction over the selected first area of Lioni.

Figure 8 and Figure 9 show the segmentation and classification of the first Lioni area used for testing the proposed algorithm.

To evaluate the accuracy of the buildings’ detection, the classification results have been compare with CTR and orthophotos, in a GIS environment, but before analyzing the results, it is important to make some considerations.

The orthophotos and the CTR used for the validation refer to two different years, 2011 and 2004, respectively. As already explained, this is usually done, because depending on the specific Italian region, the available public data are often not recent. Therefore, in the specific case, some buildings present in the orthophotos were not present in the CTR, because of new construction. On the other hand, some buildings in the CTR were demolished before the orthophotos survey, and therefore were not present in the 2011 data. For these reasons, both data have been considered as reference data for the validation. Moreover, the total number of buildings, assumed as ground truth, has been considered equal to the sum of the buildings in the CTR (excluding those demolished) and the new buildings from the orthophotos. The total number of classified buildings has been instead calculated as the sum of the correctly classified buildings and the objects incorrectly classified as buildings (false positives).

The results of the classification and the references data have been reported in a confusion matrix from which the Producer Accuracy (PA) and the User Accuracy (UP) have been calculated.

PA has been calculated by the ratio of the correctly classified buildings and the total number of buildings. UP has been calculated by the ratio of the correctly classified buildings and the total number of classified buildings.

The developed strategy, performed first on the tested area, led to detection of 200 correctly classified buildings and 14 false positives, while the amount of buildings in the reference data was 220, by resulting into a PA and PU of 91% and 93.5%, respectively, as easily retrieved from the confusion matrix represented in the Table 2.

**Figure 10:**
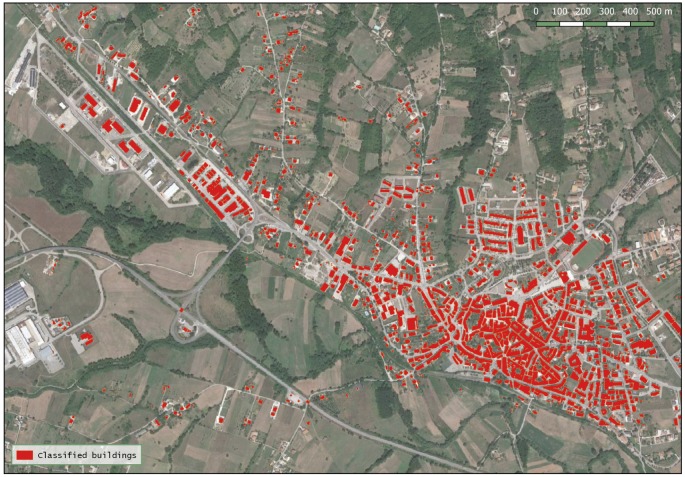
The WorldView-2 image and results of the proposed buildings extraction algorithm over Lioni town.

Figure 10 shows the results of the developed rule-set building extraction algorithm on the entire municipality of Lioni.

The amount of buildings in the reference data is 1538 and the objects classified as buildings were 1479 (1372 correctly classified and 107 false positives). Therefore, from the confusion matrix, as represented in the Table 3, PA and PU for the second area are 89% and 93%, respectively.

**Figure 11:**
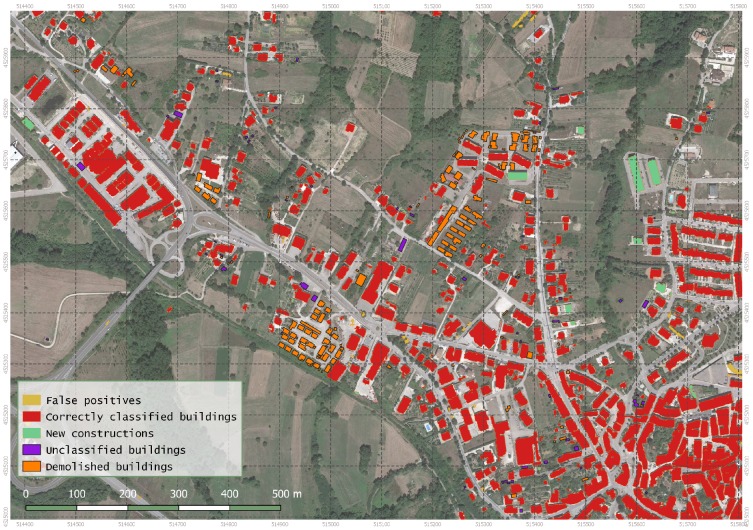
Example of updated municipal map and database.

**Figure 12:**
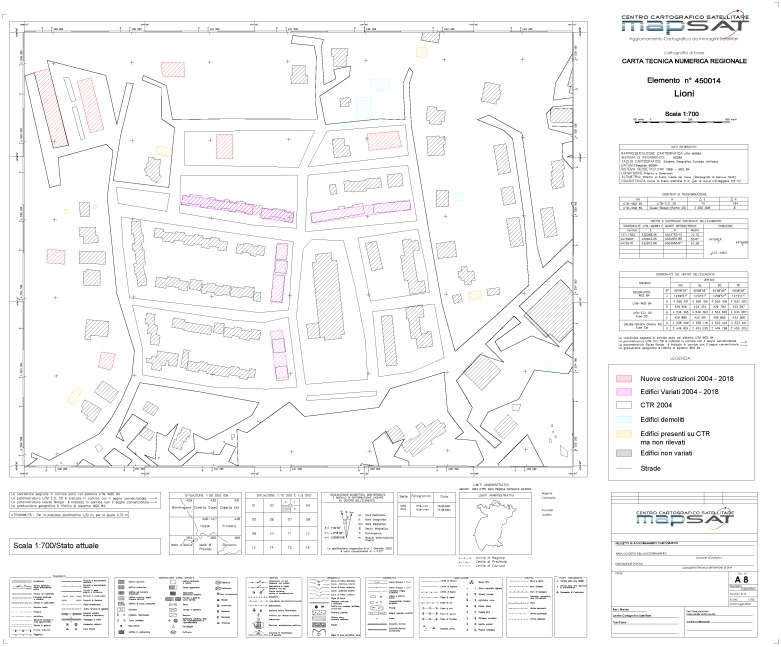
Updating of the CTR based on the results of proposed algorithm.

The low density of LiDAR points may have affected the refining of the edges of the buildings. Yet, the developed strategy remains valid since it provides new information at urban scale. In fact, the results can be used to produce or update a municipal database as shown in the Figure 11 and it can be used also for updating the CTR as shown in the Figure 12.

Also, to give an idea of the amount of resources required for running this algorithm for building extraction, computational cost (Table 4) and computer characteristics (Table 5) have been registered. However, the time complexity depends on the computer that is used and on the considered area.

The obtained results have demonstrated how the use of the LiDAR data, through the OBIA technique, can allow to obtain useful information concerning the urban land cover. Moreover, the low density of LiDAR points did not significantly affect accuracy in the algorithm developed. Furthermore, the development of a rule-set mainly based on objects information (such as height and slope), obtained from the LiDAR survey, has made possible to transfer the proposed approach to study areas of different sizes. Note that, in applying the proposed algorithm to the second area of the whole Lioni city, the rule-set has not been modified and parameters have not been adjusted. In addition, the LiDAR data used in this case are free-of-charge, because they were downloaded from public databases or they were collected through direct measurement acquisitions, as in the second Case Study presented in the next section. Unfortunately, this is the reason why one has to settle for the low density of the LiDAR point cloud in the case of public databases. Yet, it has been demonstrated that good performances have been achieved in any case, if results from the extended literature presented are used for comparison.

Future work will use more information from LiDAR data, for example, taking into account also the intensity, in order to further improve the building extraction on large areas, only using free-of-charge LiDAR data.

## 5. External 3D Scanning with a LiDAR Located at a Low Altitude

**Figure 13:**
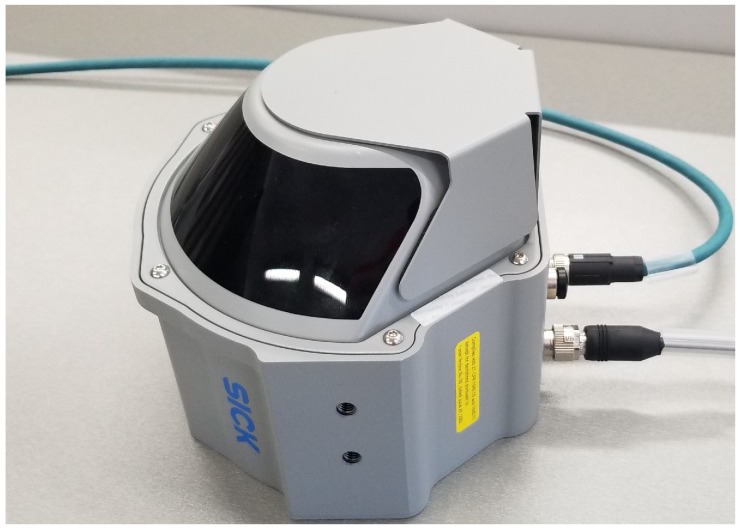
3D LiDAR MRS 6000 by the SICK company.

In this section, the objective is to show how 3D LiDARs can further help in monitoring the surrounding environment, and in identifying objects, such as buildings, trees, roads, etc. In this second case study, 3D LiDARs and other devices are presented and tested. Among them, the 3D LiDAR MRS 6000 by the SICK company, shown in Figure 13, is a very useful tool for research purposes. It will be referred to as MRS 6000 or simply LiDAR later in this section.

**Figure 14:**
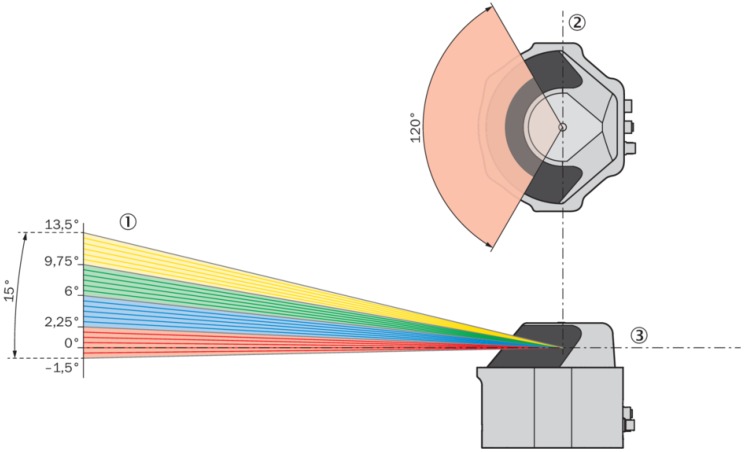
MRS 6000 range of scanning. Source: https://cdn.sick.com.

**Figure 15:**
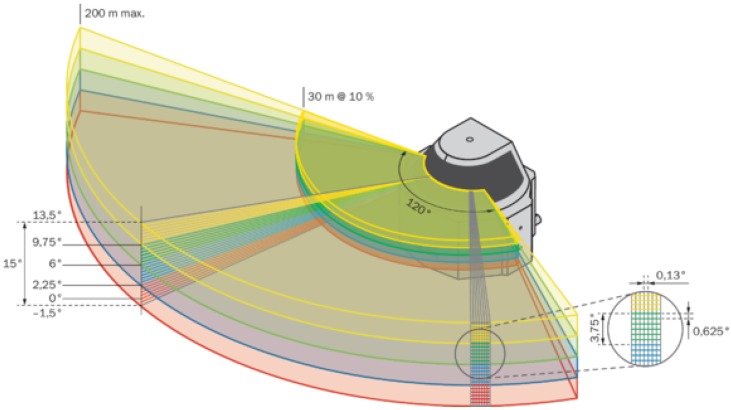
MRS 6000 range of scanning. Source: https://cdn.sick.com.

MRS 6000 has a multilayer sensor able to scanning simultaneously in all the layers. This device is an optoelectronic LiDAR sensor that uses a 24-layer non-contact laser beam, as shown in Figure 14, with a frequency of 10 Hz, which makes a 3D point cloud and creates an accurate representation of its surroundings in real-time. A unique mirror technology makes MRS 6000 a sensor that has a high scanning field stability. Using a Polygon Mirror, the LiDAR generates a straight scan field line without distortion. The LiDAR provides nearly gap-free detection through the ability to generate up to 880,000 measurement points per second (mpps). In this way, a scanning field, created on the distance of 25 meters from a sensor, contains 72 ppsm, throughout an entire aperture angle of 120 degrees horizontally and 15 degrees vertically. With a scan point density at 0.13 degrees horizontally and 0.625 degrees vertically, the distance between two individual measuring points is thereby 4.3 millimeters. LiDAR has a minimum range of 0.5 meters and a maximum range of 200 meters, but only 30 meters range at 10 percent of emission (Figure 15). In the sensor, a multi-echo evaluation is applied for increasing reliability. The LiDAR uses a calculation of time-in-flight of the emitted pulse to set up distance between the sensor and an object. Multi-echo characteristic allows to evaluate up to four echo signals for each measuring beam, and this helps in working under unfavorable weather or adverse environmental conditions, like fog, rain, or dust in the air. The data interface is 1 Gb/s Ethernet with a web app embedded.

**Figure 16:**
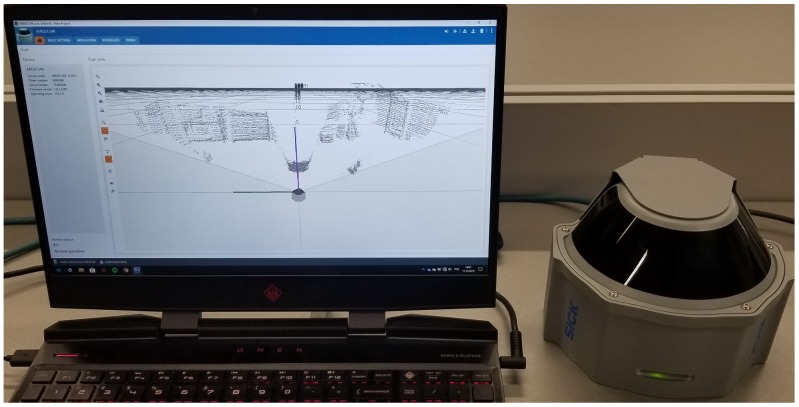
MRS 6124R testing with SOPAS ET software.

Besides the use of the MRS 6000 LiDAR, additional scans of the earth’s surface may be supplemented through the use of small UAVs moving at the height of several meters above the ground. They may allow scans by using high-resolution multilayer LiDAR that provides detailed information about objects in the area of interest. During the test, scans of the surface were made using a multilayer LiDAR and a photo of the area covered by the scan. Another Sick device, the MRS 6124R LiDAR, was used and the SOPAS ET software for visualization of the measurements was employed (Figure 16).

Scans and photos were taken from a height of 9 meters, which may correspond to the measurement from the sensors of a low-flying drone. The main idea of the tests was to determine the quality of the scan by assessing the measurement of range in low-altitude scans almost parallel to the earth’s surface. Such experiments were to imitate the measurement from a drone flying at a low altitude. Another reason was to learn about the possible form of object visualization in the acquired scan. Particular attention was paid to building identification, which is the essential task in this paper. Some Test Areas at the premises of MUT (Military University of Technology) in Warsaw, Poland, have been selected.

**Figure 17:**
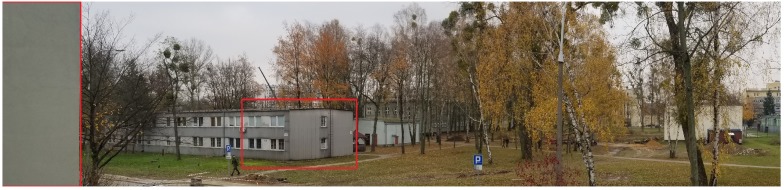
Panoramic picture of the Test Area 1.

**Figure 18:**
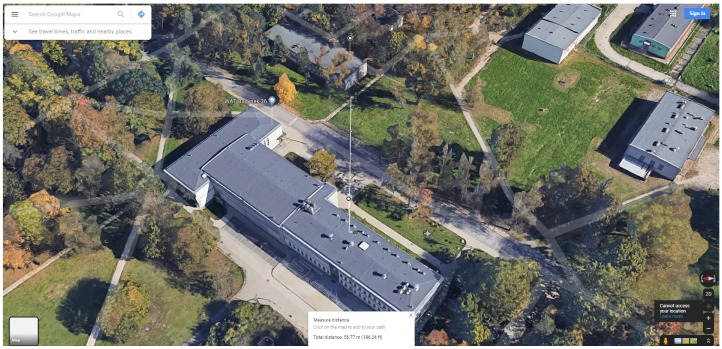
Google Maps view of the Test Area 1 with the marked distance.

**Figure 19:**
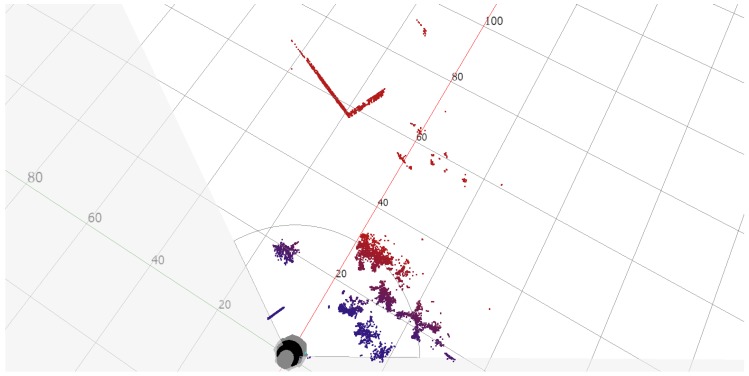

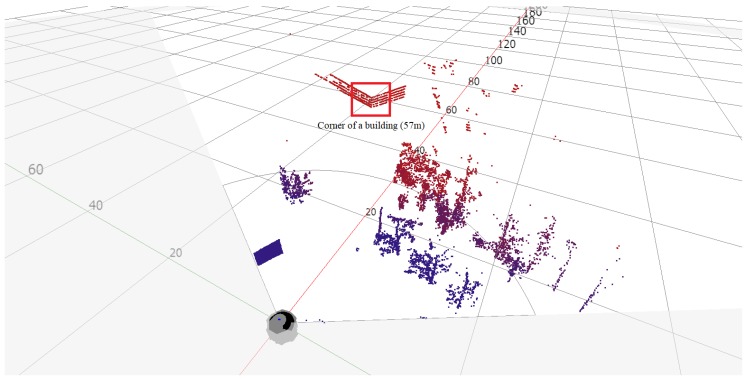
Scan of the Test Area 1.

In the Test Area 1, the reference point was a corner of the building located 57 meters from the scanner (Figure 17). The distance was determined using a laser rangefinder and shown on the image retrieved from Google maps (Figure 18). The building’s wall 8 meters away from the scanner and clearly visible in the scan foreground was also marked. It was found that the building elements are well identifiable, and the distances designated to the characteristic points of the building are consistent with the actual values (Figure 19).

**Figure 20:**
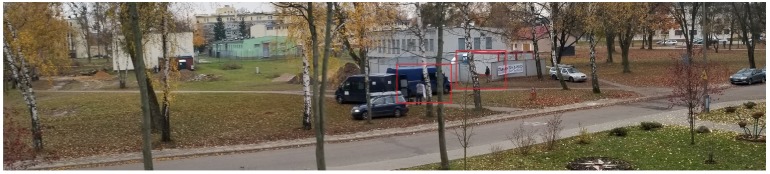
Panoramic picture of the Test Area 2.

**Figure 21:**
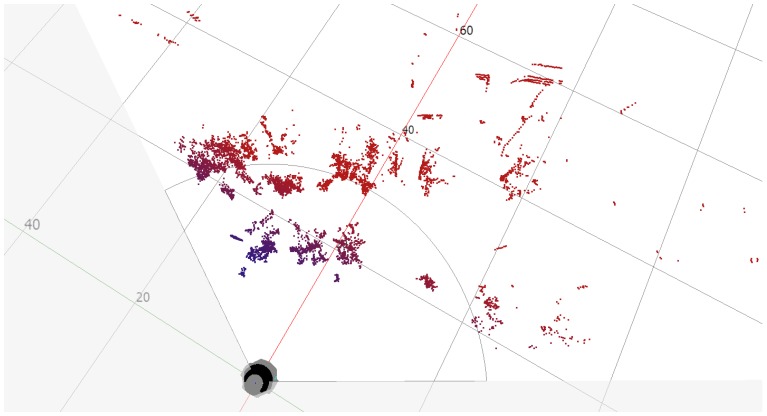

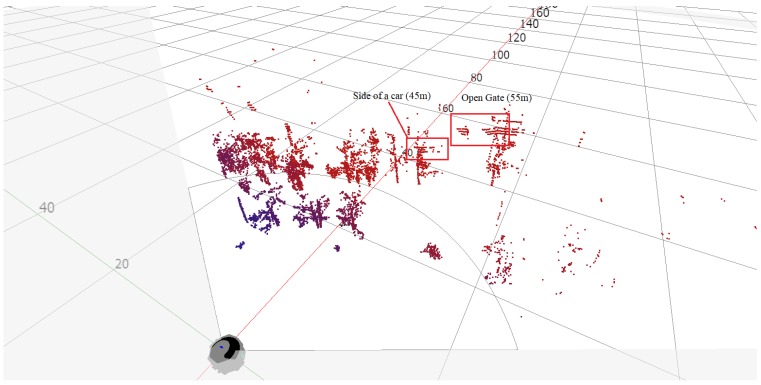
Scan of the Test Area 2.

In Test Area 2, the construction site buildings are located between 50 and 70 meters from the scanner. Cars are parked at the construction site. The gate and fence of the construction site are made of metal painted with matte paint. One of the vehicles (blue) has a matte paint. Other cars have metallic paint (Figure 20). The scans show the construction site fence and the open gate. The side of the blue van is also visible on the scan. The other two cars with metallic paint are not visible on the scan (Figure 21). On both scans, you can also see how the trees are visible, which, in this case, can be considered noise.

Analysis of scans and the corresponding photos showed that the laser beam is almost wholly deflected on reflective surfaces. As a result, reflective objects are partially mapped or utterly invisible on the scan. Objects visible through the glass are correctly mapped on the scan unless other objects are reflected in the glass. In this case, the mapped objects are superimposed on the mapped objects behind the glass. On rough surfaces, part of the energy is lost due to shadowing. Curved surfaces produce instead a higher diffusion, and finally dark surfaces reflect the laser beam worse than clear ones. Note that the surface characteristics reduce the scanning range of the device, in particular for surfaces with low emission values. This second Case Study has allowed demonstrating how 3D LiDARs can be applied to analyze the external environment by further helping in discriminating the several objects present there, with respect to 2D LiDARs. Specific devices by Stick have been used for the tests by showing that they perform correctly, despite the presence of objects, such as cars, trees, and others, that can be regarded as “noise”.

## 6. Conclusions

In this paper, we aimed to present a detailed analysis of LiDARs systems by pointing out the performance that can be achieved through the use of LiDAR data when combined with data from other sources. Two Case Studies, based on the use of 2D and 3D LiDARs (2- and 3-Dimensional LiDARs), have been presented and analyzed in an extensive way.

The Case Studies have allowed to show how the LiDAR-based systems succeed in improving the analysis and monitoring of specific areas of interest, specifically how LiDAR data can help in exploring external environment and extracting building features from urban areas. Moreover, the Case Study of Lioni has demonstrated that the use of the free-of-charge LiDAR data, even with a low density of points, allows the development of an automatic procedure for accurate building features extraction, through object-oriented classification techniques, therefore once again underlining the importance that even simple LiDAR-based systems play in EO and Environmental Monitoring.

## Figures and Tables

**Table 1:** Categories and features chosen for the building extraction rule-set.

Average Band Values	Geometric Features	Spectral Indexes
Mean (NIR1)	Area (pxl)	
Mean (Red)	Area (m${}^{2}$)	$NDVI=\frac{Mean(NIR1)\;-\;Mean(Red)}{Mean(NIR1)\;+\;Mean(Red)}$
Mean (nDSM)	Lenght/Width	
Mean (nDSM smoothed)		
Mean (Slope)		

**Table 2:** Confusion matrix.

	Result of Building Extraction			
		**Buildings**	**Other**	**Total**
**Reference data**	**Buildings**	200	20	220
	**Other**	14	0	14
	**Total**	214	20	234

**Table 3:** Confusion matrix.

	Result of Building Extraction			
		**Buildings**	**Other**	**Total**
**Reference data**	**Buildings**	1372	166	1538
	**Other**	107	0	107
	**Total**	1479	166	1645

**Table 4:** Computational cost.

Main Steps of Building Extraction	Sw	Mean Computation Time
Pre-processing	QGIS	5:15.376 (5 min and 15.376 s)
Processing	eCognition	21:59.784 (21 min and 59.784 s)

**Table 5:** Computer characteristics.

Processor	Ram	System Type
Intel(R) Core(TM) i3 CPU 530 @2.93 GHz	4.00 GB (2.99 GB available)	32-bit operating system

## Appendix A

As anticipated in Section 4.1.4, in this appendix, the detailed steps of the proposed algorithm are presented and discussed in detail.

After the creation of the smoothed nDSM, the first necessary step is to divide luminous and dark objects through a threshold that maximizes the contrast of luminous objects (consisting of pixel values above the fixed threshold) and dark objects (consisting of pixel values below the threshold).

This choice is made because the nDSM raster has high brightness in correspondence of the regions that have a certain elevation. Therefore, through the “Contrast Split Segmentation” on nDSM, it is possible to separate high objects and low objects.

After the first segmentation on the smoothed nDSM, all the objects with a value of Mean (nDSM smoothed) layer lower than 2 m are assigned to the class *Ground*, to exclude in the following steps all the objects that certainly do not belong to the building class. The remaining objects are assigned to the class *NoGround*.

Another Contrast Split Segmentation has been carried out on nDSM slope, as the generated slope layer from LiDAR data has strong contrasts of brightness on the edge of the buildings and the steep and flat areas are easily recognizable: the flat areas are associated with a very dark color, whereas the steep areas are tending to white. As reported also in other studies [56,57], the segmentation is based on the information of slopes related to the real footprint of the buildings.

All above-ground artificial objects provide a strong and sudden change in height, forming a crisp edge around them. Therefore, to fully encompass the outer building outline, the slope map is been reclassified into two classes, representing weak and strong edge in accordance to [57].

In particular, two passages of segmentation are necessary on the nDSM slope: one to obtain the objects with a strong slope, and a second one to separate those with an average slope from the very steep ones.

Therefore, after this second segmentation, based on the slope layer of objects classified as *NoGround*, all those with an average slope lower than $15^{\circ }$ are assigned to the class *NoSteep areas*, because all those objects are characterized by a weak edge. The remaining objects are assigned to the class *Steep areas*. Next, all objects with an average slope grater than $30^{\circ }$ are assigned to the temporary class *Probable buildings*. From the *Probable buildings* class, all the objects with an area with a number of pixels less than 4 (too few to be considered buildings), are excluded.

The next segmentation algorithm is the “Multiresolution segmentation”, that groups areas of similar pixels (pixels with equal values) into objects. Consequently, homogeneous areas result in larger objects, heterogeneous areas in smaller ones. The “Multiresolution segmentation” algorithm joins together existing pixels or image objects. This type of segmentation in this study was realized by exploiting the information related to the WorldView-2 image bands to create objects characterized by a strong spectral similarity, to be used in the further classification phases.

Over the objects classified as *Probable buildings*, a multi-resolution segmentation is carried out in order to create a final class *Buildings*, and to improve the geometry of the buildings. To assign the objects to the final class of *Buildings*, the height has been considered again, and a further consideration has been undertaken, based on the NDVI. The high objects, probable buildings, with a high value of NDVI are considered as *Other Elevated objects*, as they are very likely to be trees.

Moreover in the final class, buildings with an area less than 14 square meters are not considered in the analysis. Therefore garages or smaller sheds are excluded from the classification, in order to avoid misclassification errors.
